# Robotic vs. laparoscopic TAPP: a systematic review and meta-analysis of randomized controlled trials on short-term outcomes

**DOI:** 10.1007/s10029-025-03550-1

**Published:** 2025-12-12

**Authors:** Francesco Brucchi, Richard Sassun, Sara Lauricella, Roberto Cirocchi, Gianfranco Parati, Gianlorenzo Dionigi, Filip Muysoms

**Affiliations:** 1 University of Milan, Milan, 20122 Italy; 2 Colorectal Surgery Unit, Fondazione IRCCS Istituto Nazionale Dei Tumori, Milan, 20133 Italy; 3 Department of Digestive and Emergency Surgery, “S. Maria” Hospital, Terni, 05100 Italy; 4 Department of Medicine and Surgery, University of Perugia, Perugia, 06129 Italy; 5 Department of Cardiology, Istituto Auxologico Italiano, IRCCS, Milan, Italy; 6 Division of Surgery, Istituto Auxologico Italiano, Istituto di Ricovero e Cura a Carattere Scientifico (IRCCS), Milan, Italy; 7 Department of Pathophysiology and Transplantation, University of Milan, Milan, Italy; 8 Department of General Surgery, AZ Maria Middelares, Ghent, Belgium

**Keywords:** Inguinal hernia, Robotic-assisted repair, Laparoscopic TAPP, Randomized controlled trial, Meta-analysis, Systematic review, Operative time

## Abstract

**Background:**

Robotic-assisted transabdominal preperitoneal (r-TAPP) inguinal hernia repair is increasingly adopted, yet its short-term advantages over conventional laparoscopy remain uncertain.

**Methods:**

This systematic review was reported according to PRISMA guidelines. A comprehensive search was conducted in MEDLINE, Embase, and CENTRAL until September 25th, 2025. Randomized controlled trials (RCTs) comparing r-TAPP and laparoscopic TAPP were eligible. Primary outcomes were operative time and postoperative complications. A random effects model was used for meta-analysis, and study quality was assessed using the Cochrane RoB II tool.

**Results:**

Three RCTs comprising 300 patients were analyzed. Robotic repair was associated with a longer operative time, though this did not reach statistical significance (MD + 17.6 min; 95% CI − 20.7 to + 55.9; *p* = 0.37). Complication rates were not significantly different (RR 0.83; 95% CI 0.34–2.03; *p* = 0.68). Readmissions were rare and comparable between groups (RR 0.71; 95% CI 0.09–5.58; *p* = 0.74).

**Conclusions:**

Robotic TAPP is safe and effective; however, clear superiority over laparoscopy has not been established. Large-scale, multicenter RCTs with standardized protocols, long-term follow-up, and cost-effectiveness analyses are needed to clarify the role of robotics in inguinal hernia repair.

**Prospero registry:**

Registration number: CRD420251157847

**Supplementary Information:**

The online version contains supplementary material available at 10.1007/s10029-025-03550-1.

## Introduction

Inguinal hernia repair is among the most frequently performed surgical procedures worldwide, with over 20 million operations each year [1, 2]. In recent decades, the technique has evolved from open repair to minimally invasive approaches, initially laparoscopic [3] and more recently robotic surgery [4], aiming to accelerate recovery, reduce both acute and chronic postoperative pain, minimize surgical site–related complications, and ultimately improve patient quality of life [5].

Robotic platforms were introduced to overcome the technical limitations of laparoscopy, offering a stable three-dimensional view, wristed instruments that replicate natural hand movements, and enhanced precision. Despite these theoretical advantages and increasing adoption, robust evidence of clinical superiority remains lacking, while higher costs compared with laparoscopy are consistently reported [6–9].

Previous systematic reviews [6, 9–11] have shown similar complication rates but longer operative times for robotic repair, findings that may have been confounded by the inclusion of cases performed during surgeons’ learning curves. Notably, no meta-analysis to date has focused exclusively on randomised controlled trials (RCTs), which provide the highest level of evidence for comparing surgical approaches.

This systematic review and meta-analysis synthesises RCT evidence to evaluate short-term outcomes of robotic versus laparoscopic TAPP inguinal hernia repair, with particular focus on operative time and postoperative complications.

## Methods

### Search strategy

The study was conducted following the guidelines outlined in the Preferred Reporting Items for Systematic Reviews and Meta-Analyses (PRISMA) statement [12]. The research protocol was registered with the International Prospective Register of Systematic Reviews (PROSPERO) under registration number CRD420251157847 (http://www.crd.york.ac.uk/PROSPERO). A systematic search of the peer-reviewed literature published from January 1st, 2000, to September 25th, 2025, was conducted using the PubMed, Embase, Scopus, and Cochrane Library databases. Search strategies for each database were developed using various combinations of keywords (detailed in Supplementary Materials, Fig. 1s). Additional references were identified by manually screening the bibliographies of retrieved articles, systematic reviews, and meta-analyses.

**Fig. 1:**
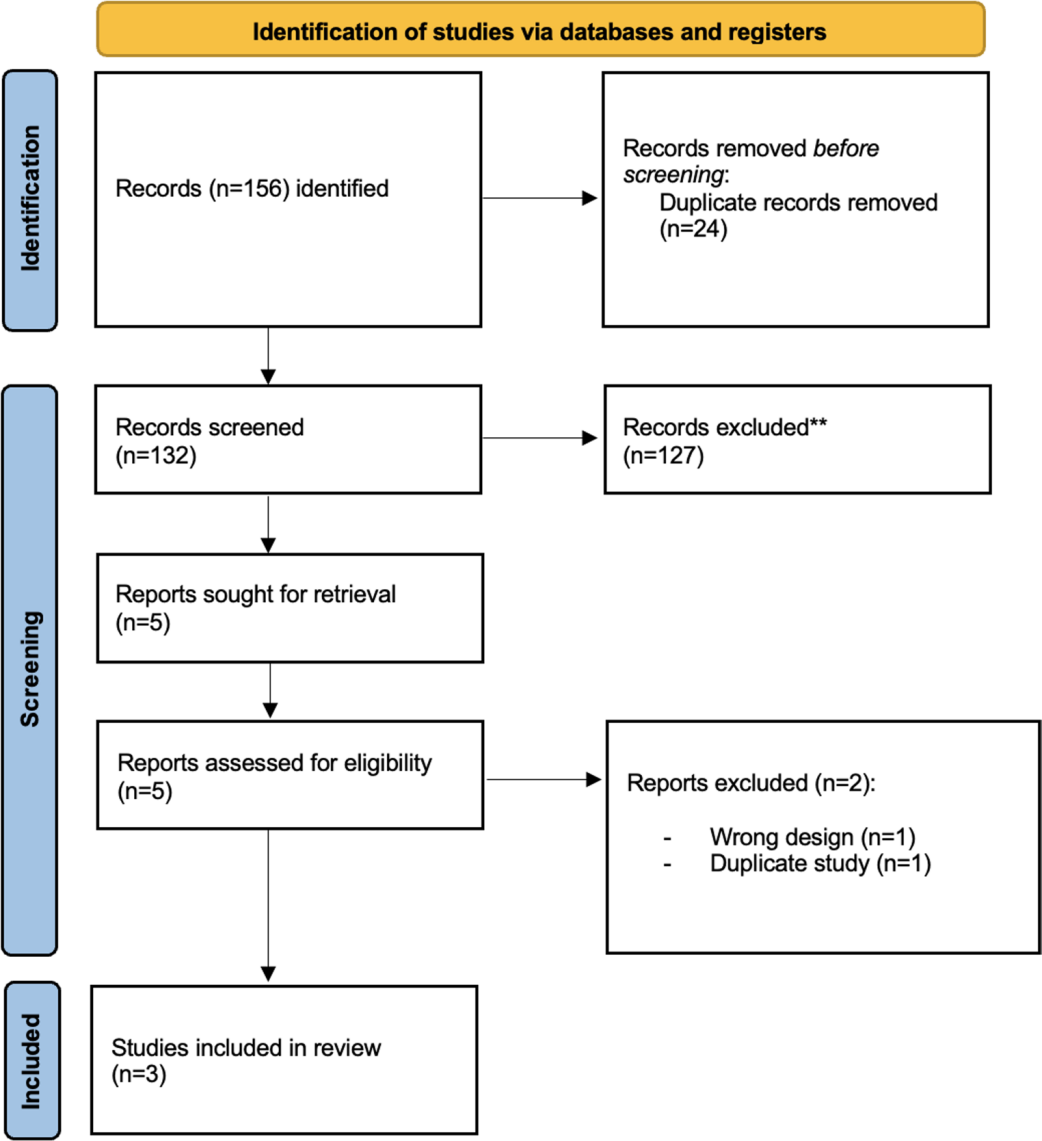
Flowchart of study screening according to PRISMA guidelines

### Study selection

Two investigators (FB, RS) independently performed the literature search and data extraction using Rayyan systematic review software. They assessed the eligibility of all preliminarily identified records independently, first based on the title and then on the abstract. After the preliminary selection, the full-text manuscripts of relevant studies were carefully reviewed to confirm eligibility and to extract useful information. Any disagreements regarding eligibility were resolved by a third reviewer (FM). Studies were included based on the following criteria: (1) randomized controlled trials written in English; (2) adult patients (≥ 18 years) undergoing inguinal hernia repair (Population) using robotic-assisted transabdominal preperitoneal (r-TAPP) repair (Intervention), compared to laparoscopic transabdominal preperitoneal (l-TAPP) repair (Control); (3) reporting at least one short-term outcome, including operative time and/or perioperative complications (Outcome).

No geographic or publication date restrictions were applied. Non-randomized studies, conference abstracts, reviews, and papers reporting duplicative results from the same author group were excluded.

### Data extraction

Two authors independently examined the main features of each included article and extracted the following data: first author, year of publication, country of origin, study period, study design, number of participants, patient demographics (age, gender, BMI, ASA score), and surgical details (operative time, hernia type, laterality, and rate of bilateral repairs). Clinical outcomes, including perioperative complication rates (minor and major, classified according to the Clavien–Dindo system), conversion to open surgery, length of hospital stay, and readmission rates, were also recorded. Data from all relevant tables, figures, and supplementary materials in the included studies were reviewed for completeness.

### Risk of bias

The methodological quality and risk of bias for each study were assessed using the Revised Cochrane Risk of Bias Tool for Randomized Trials (RoB2) [13]. To evaluate the quality of evidence for each outcome, the Grading of Recommendations Assessment, Development, and Evaluation (GRADE) approach was used, categorizing the evidence level as ‘very low,’ ‘low,’ ‘moderate,’ or ‘high.’ The quality of the evidence was downgraded or upgraded based on risk of bias, inconsistency, indirectness, imprecision, publication bias, large magnitude of effect, dose-response, or the influence of plausible confounders [14]. Two authors independently performed the GRADE assessment, and disagreements were resolved through discussion.

### Outcome measures

Primary outcomes were:

- Operative time (minutes).
- Postoperative complications (classified according to the Clavien–Dindo system [15])

Secondary outcomes were:

- Postoperative pain and analgesic requirement.
- Length of hospital stay.
- Readmission rate.

Subgroup analyses were conducted where possible, particularly examining unilateral vs. bilateral repairs, and complex vs. primary hernias.

These secondary endpoints were predefined in the review protocol; however, they were extracted and analyzed only when reported by the included randomized trials. Outcomes not available or insufficiently reported in the primary studies were not synthesized quantitatively and were presented narratively or not reported.

### Outcome definitions

Because randomized trials may use heterogeneous definitions and timepoints, all outcomes were extracted exactly as reported in each study and harmonized according to a predefined approach.

#### Operative time

Operative duration was extracted according to the definition used in each study. When trials reported a single value for total skin-to-skin time, this measure was used directly and considered the primary operative-time metric for the review. When only phase-specific times were available, total operative time was calculated as the sum of all intraoperative phases as defined by the authors, in order to obtain the skin-to-skin time. Docking time was included only when explicitly part of the study’s operative-time definition.

#### Postoperative complications

Postoperative complications were extracted at 30 days, using the definitions and classification systems employed by each original study, including any reported severity grading (e.g., Clavien–Dindo). For quantitative synthesis, complications were dichotomized as “any vs none” without reclassification of individual events.

#### Postoperative pain

Pain outcomes were extracted using the scale and scoring range employed in each trial (e.g., VAS 0–100 mm, NRS 0–10) and reported at study-specific postoperative timepoints. Owing to heterogeneity in measurement instruments and timing, pain was planned for descriptive synthesis only.

#### Length of hospital stay

Length of stay was recorded as reported in each study. Because units and definitions varied (hours, calendar days, or same-day discharge rate), LOS was prespecified for narrative reporting without quantitative pooling.

#### Readmissions

Thirty-day readmission rates were extracted as reported in each study at the predefined postoperative follow-up.

### Statistical analysis

Continuous variables were analyzed using the mean difference (MD) and 95% confidence intervals (CI). Categorical variables were evaluated using odds ratios (OR) and 95% CI. When data were reported as median and interquartile range (IQR), they were converted to mean and standard deviation (SD) using the method of Hozo et al. [16] Pooled estimates were calculated using random-effects models (DerSimonian–Laird method). Heterogeneity was assessed using the Q statistic and the I² statistic, with I² values of 25%, 50%, and 75% considered low, moderate, and high heterogeneity, respectively.

When high heterogeneity was detected, sensitivity analyses were performed by systematically excluding individual studies to assess their impact on pooled estimates. Statistical analyses were conducted using Python (Python Software Foundation, http://www.python.org).

## Results

### Study selection and quality assessment

The systematic literature search identified 156 articles through PubMed, Scopus, and Cochrane databases. After removing duplicates, 132 unique articles were screened. Based on title and abstract, 127 articles were excluded for irrelevance. Five full-text articles were assessed, of which two were excluded. Ultimately, three randomized controlled trials (RCTs) met the inclusion criteria [8, 17, 18] (Fig. 1). A summary of study characteristics is provided in Table 1. The risk of bias assessment using the RoB2 tool is presented in Supplementary Figure S1, showing an overall low risk of bias. GRADE evaluation rated the evidence as moderate to high quality for the primary outcomes (Supplementary Table S2). For the ROLAIS trial (Valorenzos et al. [18]), additional baseline and outcome data not reported in the primary publication were retrieved from the DIRECT post-hoc analysis (Arunthavanathan et al. [19]), which was conducted on the same dataset. No patients were double-counted, and information was only used to complete missing variables.

**Table 1:** Selected studies reporting the comparison between robotic and laparoscopic TAPP

First Author (Year)	Country	Study Period	Design	*n* (*r*-TAPP/l-TAPP)	Reported Outcomes
Prabhu et al. (2020) [8]	USA	2016–2019	RCT	48/54	Operative time, complications, Quality of Life, Surgeons’ ergonomics
Dixon et al. (2025) [17]	UK	2023–2024	RCT	39/20	Operative time, Surgeons’ ergonomics and strain, Non-technical skills
Valorenzos et al. (2025) [18]	Denmark	2022–2024	RCT	74/65	Operative time, complications, CRP and IL-6 levels

### Baseline characteristics

A total of 300 patients were included, with 161 undergoing robotic-assisted TAPP (r-TAPP) and 139 laparoscopic TAPP (l-TAPP). Detailed patient and surgical characteristics are summarized in Supplementary Tables S3–S5. The pooled mean age was 57.7 ± 14.7 years in the r-TAPP group and 57.3 ± 13.4 years in the l-TAPP group. Male patients accounted for 88.5% in the r-TAPP group and 87.3% in the l-TAPP group. Mean BMI averaged 25.6 ± 3.6 kg/m² in the r-TAPP group and 26.3 ± 4.1 kg/m² in the l-TAPP group.

Surgeon experience varied across trials: Prabhu et al. [8] required ≥ 25 prior robotic procedures (not r-TAPP specific), Dixon et al. [17] mandated ≥ 20 procedures in each approach, while Valorenzos et al. [18] enrolled surgeons with ≥ 100 r-TAPP and formal certification.

### Meta-Analysis

#### Operative time

**Fig. 2:**
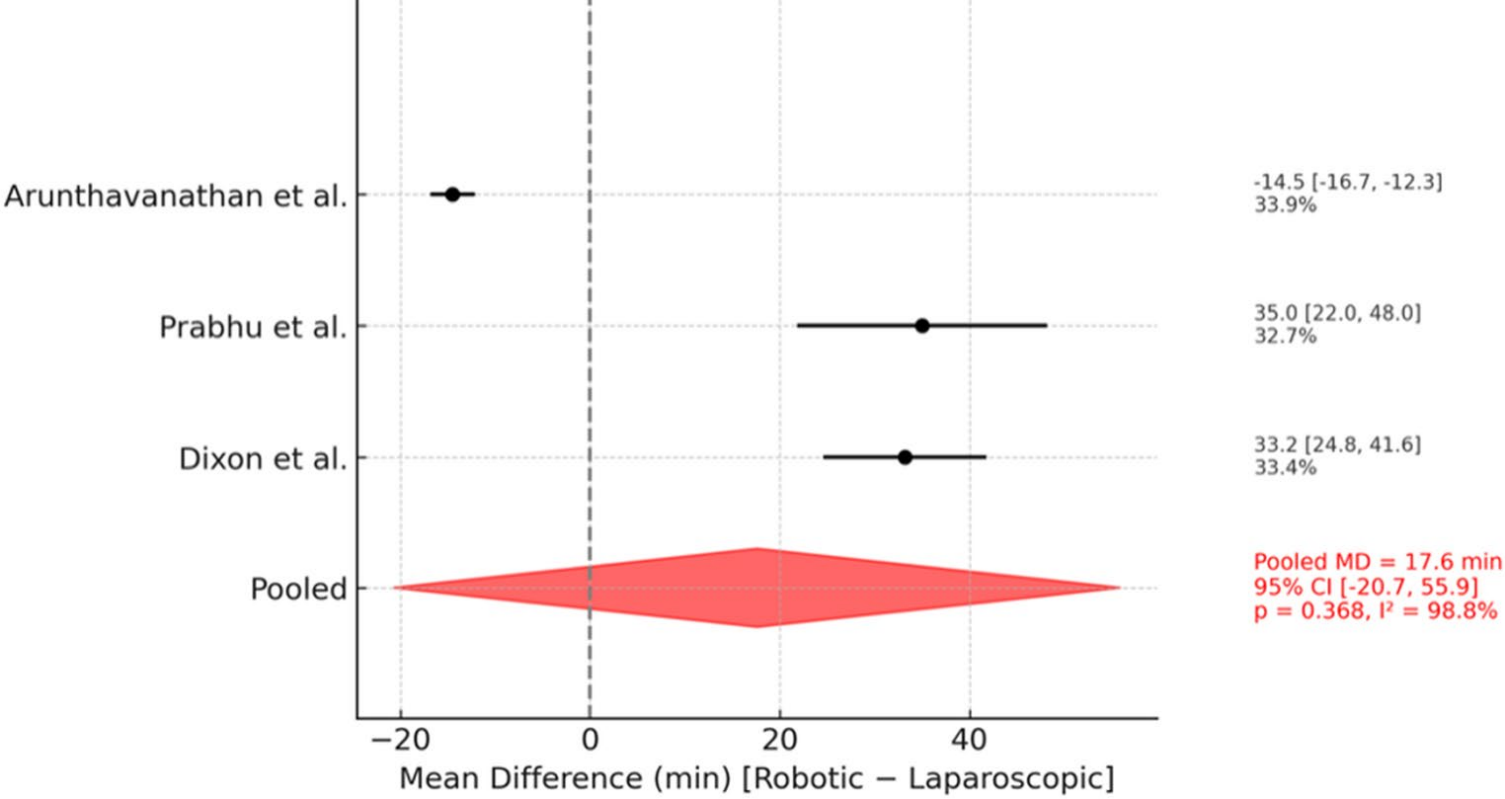
Forest plot showing operative time of robotic vs. laparoscopic TAPP

All three RCTs reported operative time [8, 17, 18]. The pooled mean operative time was 67.3 ± 17.9 min in the robotic group and 49.4 ± 13.1 min in the laparoscopic group. The random-effects meta-analysis showed no significant difference (mean difference [MD] + 17.6 min; 95% CI: −20.7 to + 55.9; *p* = 0.37; I² = 98.8%) (Fig. 2). Given the small number of trials, we did not emphasize subgroup or leave-one-out analyses; instead, we explored heterogeneity qualitatively in the discussion section.

#### Postoperative complications

**Fig. 3:**
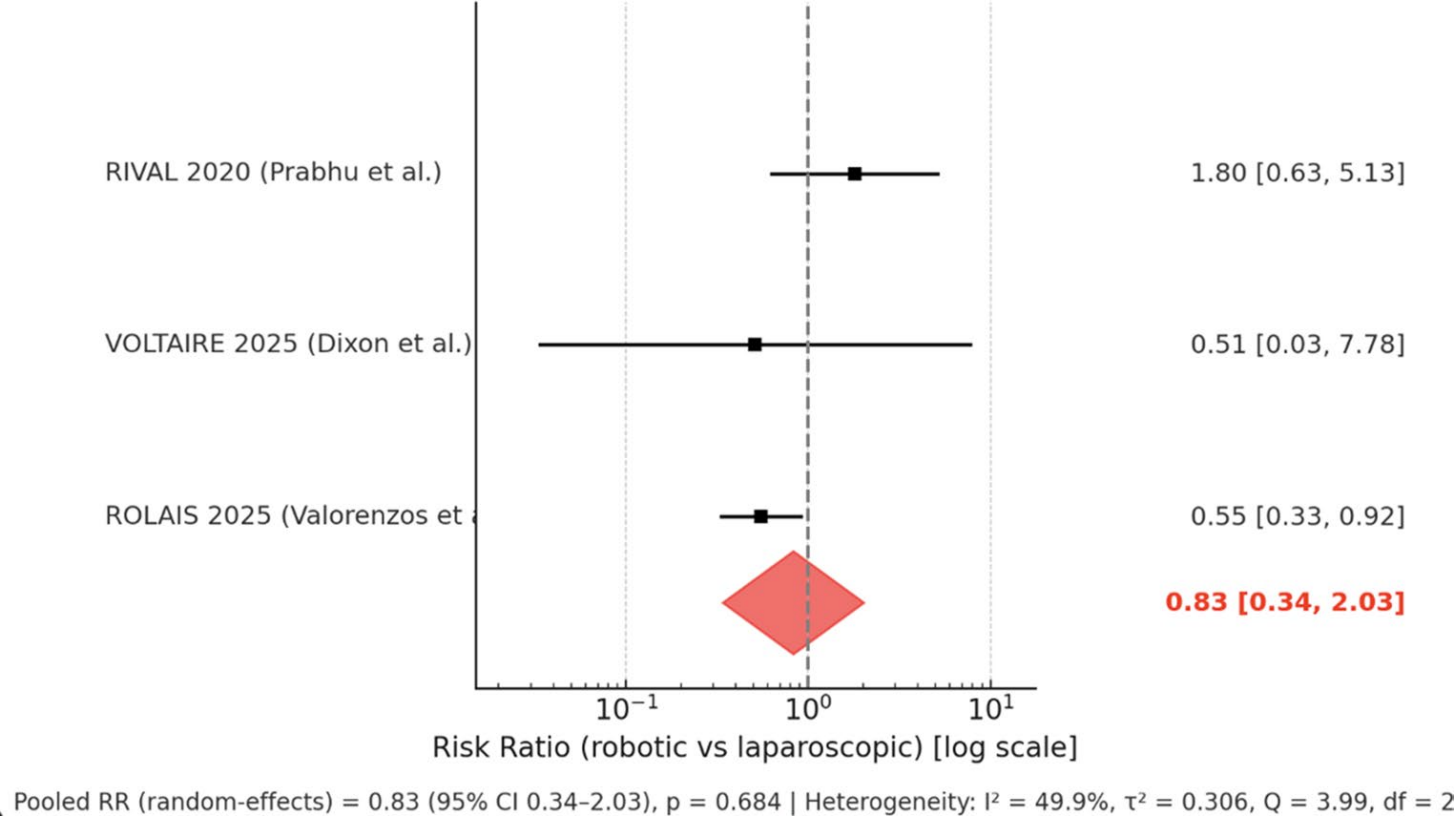
Forest plot showing postoperative complications of robotic vs. laparoscopic TAPP

All three RCTs reported postoperative complications [8, 17, 18]. Prabhu et al. [8] observed adverse events in 8/48 (16.7%) r-TAPP patients compared with 5/54 (9.3%) l-TAPP patients. Dixon et al. [17] reported 1/39 (2.6%) complications in the robotic group and 1/20 (5.0%) in the laparoscopic group. Valorenzos et al. [18] found that complications at 30 days occurred in 17/74 (23.0%) r-TAPP and 27/65 (41.5%) l-TAPP patients. When pooled across the three trials in a random-effects model, the risk ratio for any complication was 0.83 (95% CI 0.34–2.03; *p* = 0.684), with moderate heterogeneity (I² = 49.9%). These results indicate no statistically significant difference in complication rates between robotic and laparoscopic TAPP repair (Fig. 3). An overview of all complications reported across the included RCTs is provided in Supplementary Table S5.

#### Readmission rate

**Fig. 4:**
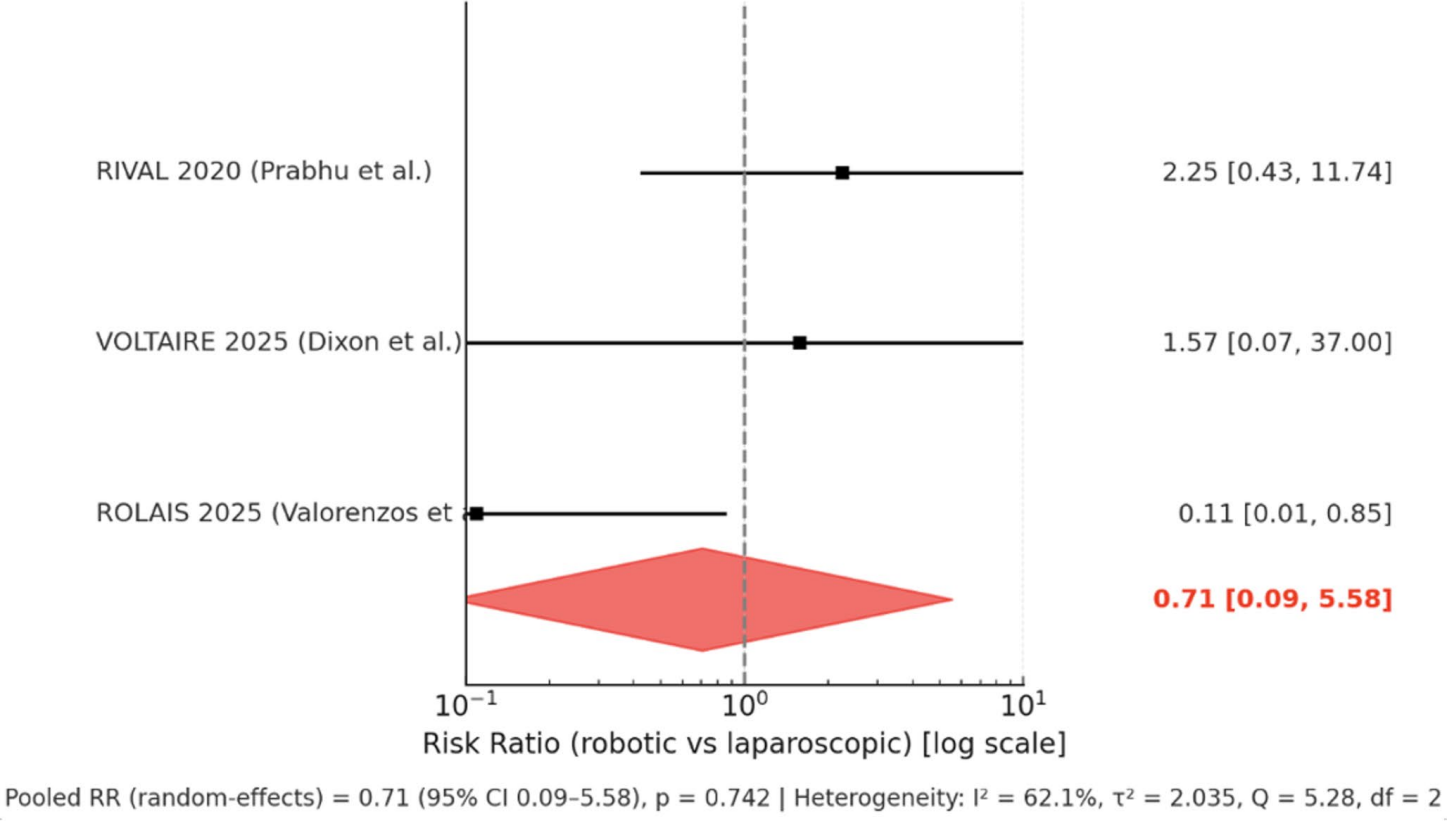
Forest plot showing 30-day readmission of robotic vs. laparoscopic TAPP

All three RCTs reported 30-day readmissions [8, 17, 18]. Prabhu et al. [8] documented 4/48 (8.3%) readmissions in the robotic group versus 2/54 (3.8%) in the laparoscopic group. Dixon et al. [17] reported 1/39 (2.6%) readmission after r-TAPP compared with 0/20 (0%) after l-TAPP. Valorenzos et al. [18] found 1/74 (1.4%) readmission with r-TAPP versus 8/65 (12.3%) with l-TAPP (*p* = 0.014). When pooled in a random-effects meta-analysis, the risk ratio for readmission was 0.71 (95% CI 0.09–5.58; *p* = 0.742), with moderate heterogeneity (I² = 62.1%) (Fig. 4).

#### Narrative synthesis of secondary outcomes

##### Postoperative pain and analgesic requirement

Two studies assessed postoperative pain [8, 17]. Prabhu et al. [8] found no significant differences in VAS scores or analgesic use at 1 week and 30 days (mean VAS at 1 week: 26.4 robotic vs. 28.9 laparoscopic). Dixon et al. [17] reported comparable pain scores on day 14 (median 0 [IQR 2] after r-TAPP vs. 1 [IQR 2] after l-TAPP; *p* = 0.46).

##### Length of hospital stay

All trials reported length of stay (LOS) [8, 18]. Prabhu et al. [8] observed a median stay of 5.8 h after robotic repair versus 5.1 h after laparoscopic repair. Valorenzos et al. [18] found same-day discharge in 95.9% (71/74) of r-TAPP patients compared with 81.5% (53/65) of l-TAPP patients (*p* = 0.006). Dixon et al. [17] reported both groups showing a median of 0 days (IQR = 1; routine same-day discharge).

## Discussion

To our knowledge, this is the first systematic review and meta-analysis exclusively restricted to randomised controlled trials, thereby providing the highest level of evidence to date regarding short-term outcomes of robotic versus laparoscopic TAPP inguinal hernia repair. Across three studies [8, 17, 18] (*n* = 300 patients), no statistically significant differences were identified in operative time, postoperative complications, or readmission rates.

The interpretation of operative time warrants particular caution. Although the pooled analysis suggested a longer mean duration for r-TAPP (67.3 vs. 49.4 min), differences did not reach statistical significance and must be contextualised by substantial heterogeneity between studies. Dixon et al. [17] utilised the Versius^®^ platform, Prabhu et al. [8] a combination of da Vinci^®^ Si and Xi systems, and Valorenzos et al. [18] exclusively the da Vinci^®^ Xi. Variations in platform type, docking time, instrument handling, and mesh fixation strategies likely contributed to the observed inconsistencies. For example, in Prabhu et al. [8], docking added only 5 min, but mesh fixation via suturing in the robotic group versus tackers in the laparoscopic group may invalidate direct time comparisons. In Dixon et al. [17], the higher BMI of patients undergoing robotic repair (27.1 ± 3.6 vs. 24.2 ± 3.5) and a lack of standardised technique further limit interpretation. Collectively, these findings indicate that operative time is a multifactorial outcome influenced by platform generation, surgical technique, and experience, rather than robotics per se. Notably, bilateral and complex hernias—known to increase operative duration—were balanced in Dixon et al. [17] and Valorenzos et al. [18] but excluded in Prabhu et al. [8] This is notable, as Arunthavanathan et al. [19], in their DIRECT trial, a post-hoc analysis of the ROLAIS dataset [18], highlighted a potential benefit of robotics precisely in such demanding cases. Other non-randomized, single-surgeon studies from high-volume centers have also reported similar results [20, 21].

Surgical expertise and the learning curve are additional critical determinants of outcomes. In Prabhu et al. [8], surgeons were required to have completed 25 prior robotic cases, but not specifically r-TAPP, while Dixon et al. [17] mandated only 20 procedures per approach, reflecting limited experience. Conversely, Valorenzos et al. [18] enrolled surgeons with ≥ 100 r-TAPPs and formal certification, effectively minimising learning-curve effects. Importantly, only this latter trial demonstrated a significant reduction in complications with robotics, underscoring the importance of structured training and substantiating the need for specialised programmes in robotic abdominal wall surgery to optimise both patient safety and resources utilization [22–24].

This meta-analysis did not show a statistically significant reduction in complications with robotics. In particular, Valorenzos et al. [18], which included the most experienced surgeons, reported a notable reduction in 30-day complications, while Dixon et al. [17] documented faster resumption of normal activities and Prabhu et al. found no differences in quality of life. The apparently higher overall complication rate reported in the ROLAIS trial (41.5% for L-TAPP vs. 23% for R-TAPP) may largely reflect the study’s comprehensive documentation of all Clavien–Dindo grade I–II events, the inclusion of data not considered in other trials (CPIP in their study was included in the postoperative complications) and the inclusion of more complex hernias (bilateral, recurrent, or inguinoscrotal), rather than indicating a true increase in morbidity. More than 85% of all complications were minor and managed conservatively. It should also be noted that all participating surgeons routinely performed both laparoscopic and robotic TAPP repairs and had completed ≥ 100 R-TAPP procedures, which makes significant skill decay in laparoscopic procedures unlikely. These aspects suggest that the observed differences are more likely related to methodological factors than to surgical proficiency.

In terms of CPIP, although preliminary data from ROLAIS suggested a numerically lower rate of this outcome after robotic repair, current RCTs are clearly underpowered for this endpoint. Future studies systematically assessing CPIP with standardized criteria could allow future meta-analyses to determine whether robotics offers a genuine advantage in this regard.

A persistent drawback is the higher cost associated with robotic repair, primarily attributable to operating room and disposable instrument expenses, with no evidence of offsetting benefits in the short term. Nevertheless, preliminary signals from high-proficiency centres suggest that clinical advantages may become apparent as the learning curve is surpassed. Surgeons also report superior visualisation and anatomical delineation with robotic systems, potentially facilitating safer dissection, although whether these advantages yield tangible patient benefits remains to be established. Overall, robotic TAPP appears to offer short-term outcomes comparable to laparoscopy, with possible incremental benefits in experienced hands that require confirmation in higher-powered studies.

Readmission rates were uniformly low and did not differ between groups. While Valorenzos et al. [18] found a statistically significant readmission reduction with robotic repair, this was not corroborated in pooled analysis, which was likely underpowered due to small sample sizes and event rates.

Several limitations must be acknowledged (Table 2). This review is based on only three RCTs with modest sample sizes, limiting power and precision. Heterogeneity was substantial, particularly for operative time, reflecting differences in platforms, surgical techniques, and baseline characteristics (e.g., higher BMI in the robotic arm of Dixon et al. [17]). Outcome definitions were inconsistent: pain was measured with different instruments, LOS variably reported, and recurrence only captured in the short term. Dixon et al. [17] and Valorenzos et al. [18] included bilateral and complex cases, whereas Prabhu et al. [8] enrolled only unilateral hernias, limiting generalizability. Learning-curve effects likely influenced results in Prabhu et al. [8] and Dixon et al. [17], while only Valorenzos et al. [18] ensured high proficiency. All trials were open-label, raising risk of bias for subjective outcomes. Finally, costs were reported in a single trial, precluding firm conclusions on cost-effectiveness.

**Table 2:** Summary of the limitations of the present systematic review and meta-analysis

Limitation	Details
Number and size of trials	Only 3 RCTs included; sample sizes are modest, limiting statistical power and effect estimate precision.
Heterogeneity	Notable differences in robotic platforms, surgical techniques, and patient characteristics (e.g., BMI) contributed to heterogeneity, especially for operative time.
Outcome definitions	Variability in measurement tools for pain, reporting of length of stay, and short-term recurrence only; inconsistent outcome definitions across studies.
Case selection	Inclusion criteria varied: Prabhu et al. studied only unilateral cases, while others included bilateral and complex hernias, restricting generalizability.
Surgical experience	Surgeon proficiency varied; learning-curve effect likely influenced results except in Valorenzos et al. with certified, experienced surgeons.
Blinding and bias	All studies were open-label, increasing risk of bias for subjective outcomes such as patient-reported measures.
Cost-effectiveness	Cost comparisons limited: only one trial reported expenses, and none included formal cost-utility analysis.
Follow-up duration	Trials had relatively short follow-up periods, limiting conclusions regarding chronic pain, long-term mesh complications, or late recurrence.
Surgeon numbers	Small number of participating surgeons with differing experience impairs external validity.

Despite these limitations, this review provides the most robust synthesis of RCT data currently available. Overall, the available evidence indicates that robotic TAPP achieves short-term outcomes comparable to laparoscopic repair, but definitive conclusions regarding its clinical and economic advantages remain elusive. Future research should move beyond feasibility studies toward large, collaborative, expertise-based randomized trials designed to critically assess the true utility, value, and long-term impact of robotic platforms in inguinal hernia surgery. It is unrealistic to expect universal standardization of platforms and techniques, but future studies should strive to report operative time by phase (docking, dissection, fixation, closure), describe the system used, and detail critical surgical steps. Rigorous methodological reporting will be essential to resolve ongoing debates regarding operative time. Until more definitive data are available, adoption of robotic TAPP should be tailored to available resources, surgeon experience, and patient preferences, with particular utility in complex cases and bilateral repairs once the learning curve has been overcome [20], according to operator expertise [19, 25] and for training purposes [24].

## Conclusion

Robotic TAPP is safe and effective; however, clear superiority over laparoscopy has not yet been demonstrated, and its cost-effectiveness remains unproven and highly context-dependent. The expanding adoption of robotic platforms underscores the urgent need for rigorous, large-scale, multicentre randomized controlled trials—stratified by hernia complexity and surgeon experience, and including comprehensive economic analyses—to determine whether their use translates into tangible patient benefit. Such coordinated, collaborative efforts are essential to generate definitive evidence while maintaining balanced training pathways that preserve laparoscopic expertise in future generations of surgeons.

## Supplementary Information

Below is the link to the electronic supplementary material.

Supplementary Material 1

## References

[1] Brucchi F, Ferraina F, Masci E, Ferrara D, Bottero L, Faillace GG (2023) Standardization and learning curve in laparoscopic hernia repair: experience of a high-volume center. BMC Surg 23:212. 10.1186/s12893-023-02119-y37507714 10.1186/s12893-023-02119-yPMC10385909

[2] Köckerling F, Simons MP (2018) Current concepts of inguinal hernia repair. Visc Med 34:145–150. 10.1159/00048727829888245 10.1159/000487278PMC5981671

[3] Brucchi F, Ferraina F, Masci E, Ferrara D, Cassini D, Faillace G (2024) To close, not to close, or to act bigger? Managing the defect of large direct inguinal hernia to reduce the risk of recurrence during laparoscopic TAPP repair: a retrospective cohort study. Updat Surg 76:2395–2402. 10.1007/s13304-024-01870-y10.1007/s13304-024-01870-yPMC1154136438733485

[4] Finley DS, Rodriguez E, Ahlering TE (2007) Combined inguinal hernia repair with prosthetic mesh during transperitoneal robot assisted laparoscopic radical prostatectomy: A 4-Year experience. J Urol 178:1296–1300. 10.1016/j.juro.2007.05.15417698133 10.1016/j.juro.2007.05.154

[5] Stabilini C, Van Veenendaal N, Aasvang E, Agresta F, Aufenacker T, Berrevoet F, Burgmans I, Chen D, De Beaux A, East B, Garcia-Alamino J, Henriksen N, Köckerling F, Kukleta J, Loos M, Lopez-Cano M, Lorenz R, Miserez M, Montgomery A, Morales-Conde S, Oppong C, Pawlak M, Podda M, Reinpold W, Sanders D, Sartori A, Tran HM, Verdaguer M, Wiessner R, Yeboah M, Zwaans W, Simons M (2023) Update of the international herniasurge guidelines for groin hernia management. BJS Open 7:zrad080. 10.1093/bjsopen/zrad08037862616 10.1093/bjsopen/zrad080PMC10588975

[6] Li X, Li Y-J, Dong H, Wang D-C, Wei J (2024) Meta-analysis of the effectiveness and safety of robotic-assisted versus laparoscopic transabdominal preperitoneal repair for inguinal hernia. PLoS ONE 19:e0298989. 10.1371/journal.pone.029898938408054 10.1371/journal.pone.0298989PMC10896538

[7] Miller BT, Prabhu AS, Petro CC, Beffa LRA, Carbonell AM, Hope W, Warren J, Higgins RM, Jacob B, Blatnik J, Krpata DM, Tu C, Costanzo A, Rosen MJ (2023) Laparoscopic versus robotic inguinal hernia repair: 1- and 2-year outcomes from the RIVAL trial. Surg Endosc 37:723–728. 10.1007/s00464-022-09320-935578051 10.1007/s00464-022-09320-9

[8] Prabhu AS, Carbonell A, Hope W, Warren J, Higgins R, Jacob B, Blatnik J, Haskins I, Alkhatib H, Tastaldi L, Fafaj A, Tu C, Rosen MJ (2020) Robotic inguinal vs transabdominal laparoscopic inguinal hernia repair: the RIVAL randomized clinical trial. JAMA Surg 155:380. 10.1001/jamasurg.2020.003432186683 10.1001/jamasurg.2020.0034PMC7081145

[9] Solaini L, Cavaliere D, Avanzolini A, Rocco G, Ercolani G (2022) Robotic versus laparoscopic inguinal hernia repair: an updated systematic review and meta-analysis. J Robot Surg 16:775–781. 10.1007/s11701-021-01312-634609697 10.1007/s11701-021-01312-6PMC9314304

[10] Ye L, Childers CP, De Virgilio M, Shenoy R, Mederos MA, Mak SS, Begashaw MM, Booth MS, Shekelle PG, Wilson M, Gunnar W, Girgis MD, Maggard-Gibbons M (2021) Clinical outcomes and cost of robotic ventral hernia repair: systematic review. BJS Open 5:zrab098. 10.1093/bjsopen/zrab09834791049 10.1093/bjsopen/zrab098PMC8599882

[11] de’Angelis N, Schena CA, Moszkowicz D, Kuperas C, Fara R, Gaujoux S, Gillion J-F, Gronnier C, Loriau J, Mathonnet M, Oberlin O, Perez M, Renard Y, Romain B, Passot G, Pessaux P The association Française de chirurgie (AFC) and the Société Française de chirurgie Pariétale - Club Hernie (SFCP-CH) (2024) Robotic surgery for inguinal and ventral hernia repair: a systematic review and meta-analysis. Surg Endosc 38:24–46. 10.1007/s00464-023-10545-510.1007/s00464-023-10545-537985490

[12] Page MJ, McKenzie JE, Bossuyt PM, Boutron I, Hoffmann TC, Mulrow CD, Shamseer L, Tetzlaff JM, Akl EA, Brennan SE, Chou R, Glanville J, Grimshaw JM, Hróbjartsson A, Lalu MM, Li T, Loder EW, Mayo-Wilson E, McDonald S, McGuinness LA, Stewart LA, Thomas J, Tricco AC, Welch VA, Whiting P, Moher D (2021) The PRISMA 2020 statement: an updated guideline for reporting systematic reviews. BMJ n71. 10.1136/bmj.n7110.1136/bmj.n71PMC800592433782057

[13] Sterne JAC, Savović J, Page MJ, Elbers RG, Blencowe NS, Boutron I, Cates CJ, Cheng H-Y, Corbett MS, Eldridge SM, Emberson JR, Hernán MA, Hopewell S, Hróbjartsson A, Junqueira DR, Jüni P, Kirkham JJ, Lasserson T, Li T, McAleenan A, Reeves BC, Shepperd S, Shrier I, Stewart LA, Tilling K, White IR, Whiting PF, Higgins JPT (2019) RoB 2: a revised tool for assessing risk of bias in randomised trials. BMJ l4898. 10.1136/bmj.l489810.1136/bmj.l489831462531

[14] Guyatt GH, Oxman AD, Vist GE, Kunz R, Falck-Ytter Y, Alonso-Coello P, Schünemann HJ (2008) GRADE: an emerging consensus on rating quality of evidence and strength of recommendations. BMJ 336:924–926. 10.1136/bmj.39489.470347.AD18436948 10.1136/bmj.39489.470347.ADPMC2335261

[15] Clavien PA, Barkun J, De Oliveira ML, Vauthey JN, Dindo D, Schulick RD, De Santibañes E, Pekolj J, Slankamenac K, Bassi C, Graf R, Vonlanthen R, Padbury R, Cameron JL, Makuuchi M (2009) The Clavien-Dindo classification of surgical complications: Five-Year experience. Ann Surg 250:187–196. 10.1097/SLA.0b013e3181b13ca219638912 10.1097/SLA.0b013e3181b13ca2

[16] Hozo SP, Djulbegovic B, Hozo I (2005) Estimating the mean and variance from the median, range, and the size of a sample. BMC Med Res Methodol 5:13. 10.1186/1471-2288-5-1315840177 10.1186/1471-2288-5-13PMC1097734

[17] Dixon F, Qureshi A, Vitish-Sharma P, Khanna A, Keeler BD (2025) Robotic assisted surgery reduces ergonomic risk during minimally invasive inguinal hernia repair: the VOLTAIRE randomised controlled trial. Am J Surg 248:116429. 10.1016/j.amjsurg.2025.11642940450465 10.1016/j.amjsurg.2025.116429

[18] Valorenzos A, Nielsen KA, Kaiser K, Petersen SR, Helligsø P, Dorfelt A, Lambertsen KL, Ellebæk MB, Nielsen MF (2025) Inflammatory response and short-term outcomes after laparoscopic *versus* robotic transabdominal preperitoneal inguinal hernia repair: randomized clinical trial (ROLAIS). Br J Surg 112:znaf074. 10.1093/bjs/znaf07440277023 10.1093/bjs/znaf074

[19] Arunthavanathan D, Liu R, Inan I, Oztoprak M, Nielsen MF (2025) Shorter operative times following robotic-assisted transabdominal preperitoneal inguinal hernia repair (TAPP) compared to laparoscopic TAPP: the Danish inguinal randomized controlled trial (DIRECT). Hernia 29:227. 10.1007/s10029-025-03402-y40632168 10.1007/s10029-025-03402-yPMC12241199

[20] Kudsi OY, Bou-Ayash N, Gokcal F, Crawford AS, Chung SK, Chudner A, Litwin D (2022) Learning curve of robot-assisted transabdominal preperitoneal (rTAPP) inguinal hernia repair: a cumulative sum (CUSUM) analysis. Surg Endosc 36:1827–1837. 10.1007/s00464-021-08462-633825019 10.1007/s00464-021-08462-6

[21] Muysoms F, Van Cleven S, Kyle-Leinhase I, Ballecer C, Ramaswamy A (2018) Robotic-assisted laparoscopic groin hernia repair: observational case-control study on the operative time during the learning curve. Surg Endosc 32:4850–4859. 10.1007/s00464-018-6236-729766308 10.1007/s00464-018-6236-7

[22] Brucchi F, Vierstraete M, Vanderstraeten E, Mottrie A, Rashidian N, Muysoms F (2025) Proposal for the hernia ASCEND Hugo^™^ RAS training pathway: acquisition of skills by comprehensive exercise-based nimbleness and dexterity training. J Robot Surg 19:516. 10.1007/s11701-025-02704-840856864 10.1007/s11701-025-02704-8PMC12380880

[23] Brucchi F, De Troyer A, Gori A, Dionigi G, Vanderstraeten E, Mottrie A, Van Herzeele I, Rashidian N, Muysoms F (2025) Structured training in robotic abdominal wall surgery: A systematic review of educational Models, Methodologies, existing gaps and unmet needs. J Abdom Wall Surg 4:15190. 10.3389/jaws.2025.1519040900686 10.3389/jaws.2025.15190PMC12399436

[24] Vierstraete M, Simons M, Borch K, De Beaux A, East B, Reinpold W, Stabilini C, Muysoms F (2022) Description of the current Da Vinci^®^ training pathway for robotic abdominal wall surgery by the European hernia society. J Abdom Wall Surg 1:10914. 10.3389/jaws.2022.1091438314150 10.3389/jaws.2022.10914PMC10831684

[25] Muysoms F, Nachtergaele F, Pletinckx P, Dewulf M (2021) ROBotic utility for surgical treatment of hernias (ROBUST hernia project). Cir Esp Engl Ed 99:629–634. 10.1016/j.cireng.2021.10.00234749923 10.1016/j.cireng.2021.10.002

