# Supplementary material for: Robotic vs. laparoscopic TAPP: a systematic review and meta-analysis of randomized controlled trials on short-term outcomes
Source: Hernia. 2025 Dec 12;30(1):36. doi: 10.1007/s10029-025-03550-1 (PMC12700930; doi:10.1007/s10029-025-03550-1)
Supplement: Supplementary file 1 — Supplementary Material 1 [file 10029_2025_3550_MOESM1_ESM.docx]

**Fig. S1 - Search Strategy**

("Hernia, Inguinal"[Mesh] OR inguinal hernia*[tiab] OR groin hernia*[tiab])

AND

("Robotic Surgical Procedures"[Mesh] OR "Robotics"[Mesh] OR robot*[tiab] OR robotic-assisted[tiab] OR robotic surgery[tiab] OR robotic TAPP[tiab] OR r-TAPP[tiab])

AND

("Laparoscopy"[Mesh] OR laparoscop*[tiab] OR minimally invasive surg*[tiab] OR laparoscopic TAPP[tiab] OR l-TAPP[tiab])

AND

("Randomized Controlled Trial"[Publication Type] OR "Randomized Controlled Trials as Topic"[Mesh] OR random*[tiab] OR trial[tiab])

**Supplementary Tables**

# **Supplementary Table S1 – Risk of Bias (RoB2) Assessment**

| First Author (Year) | Randomization Process | Deviations from Intended Interventions | Missing Outcome Data | Measurement of Outcome | Selection of Reported Result | Overall Risk |
| --- | --- | --- | --- | --- | --- | --- |
| Dixon et al. (2025) | Low risk | Some concerns | Low risk | Some concerns | Some concerns | Some concern |
| Prabhu et al. (2020) | Low risk | Some concerns | Low risk | Low risk | Low risk | Some concern |
| Valorenzos et al. (2025) | Low risk | Low risk | Low risk | Low risk | Low risk | Low risk |

**Supplementary Table S2 – GRADE Quality Assessment**

| Outcome | No. of Participants | Studies Included | Risk of Bias | Inconsistency | Indirectness | Imprecision | Publication Bias | Overall GRADE |
| --- | --- | --- | --- | --- | --- | --- | --- | --- |
| Operative time | 300 | 3 | Serious† | Serious‡ | Not serious | Serious§ | Undetected | ⨁⨁◯◯ Moderate |
| Complication rate | 240 | 2 | Serious† | Serious¶ | Not serious | Serious§ | Undetected | ⨁⨁◯◯ Moderate |
| Readmissions | 300 | 3 | Low risk | Not serious | Not serious | Serious | Undetected | ⨁⨁◯◯ Moderate |

† Due to high heterogeneity (I² > 75%) and small number of studies.

‡ High variability in results across trials.

§ Wide confidence intervals including null effect.

¶ High heterogeneity (I² = 79%).

**Supplementary Table S3 - Patient characteristics**

| **Study** | **Group** | **N** | **Age, mean ± SD** | **Male, %** | **BMI, mean ± SD** | **Notes** |
| --- | --- | --- | --- | --- | --- | --- |
| Prabhu et al. | r-TAPP | 48 | 56.1 ± 14.1 | 92 | 24.9 ± 3.2 | Only unilateral hernias included |
| Prabhu et al. | l-TAPP | 54 | 57.2 ± 13.3 | 89 | 26.9 ± 4.4 |  |
| Dixon et al. | r-TAPP | 39 | 57.4 ± 12.6 | 89 | 27.1 ± 3.6 | Stratified for unilateral/bilateral |
| Dixon et al. | l-TAPP | 20 | 58.1 ± 13.1 | 90 | 24.2 ± 3.5 |  |
| Valorenzos et al. | r-TAPP | 74 | 59.0 ± 14.0 | 91 | 27.0 ± 4.0 | Includes complex cases (27%) |
| Valorenzos et al. | l-TAPP | 65 | 60.0 ± 13.0 | 89 | 26.8 ± 3.9 | Complex cases 28% |

# **Supplementary Table S4 – Hernia Characteristics**

#

| **Study** | **Group** | **Unilateral, n (%)** | **Bilateral, n (%)** | **Complex (bilateral, recurrent, inguinoscrotal), n (%)** |
| --- | --- | --- | --- | --- |
| Prabhu et al. | r-TAPP | 48 (100) | 0 | 0 |
| Prabhu et al. | l-TAPP | 54 (100) | 0 | 0 |
| Dixon et al. | r-TAPP | 36 (92) | 3 (8) | – |
| Dixon et al. | l-TAPP | 18 (90) | 2 (10) | – |
| Valorenzos et al. | r-TAPP | – | – | 20 (27) |
| Valorenzos et al. | l-TAPP | – | – | 18 (28) |

#

#

# **Supplementary Table S5: Complication types and severity in included RCTs**

| **Study** | **Group** | **Any complications n/N (%)** | **Minor complications (Clavien I–II, where available)** | **Major complications (Clavien ≥IIIa, where available)** | **Specific events** | **Intraoperative injuries** | **Reintervention** | **Readmission (30d)** |
| --- | --- | --- | --- | --- | --- | --- | --- | --- |
| Prabhu et al. (RIVAL) | r-TAPP | 8/48 (16.7%) | Seroma, urinary retention, wound infection, hematoma (numbers NR) | NR | Seroma; urinary retention; superficial SSI; hematoma | None reported | None | 4/48 (8.3%) |
| Prabhu et al. (RIVAL) | l-TAPP | 5/54 (9.3%) | Seroma, urinary retention, wound infection, hematoma (numbers NR) | NR | Seroma; urinary retention; superficial SSI; hematoma | None reported | None | 2/54 (3.8%) |
| Dixon et al. (VOLTAIRE) | r-TAPP | 1/39 (2.6%) | 1 Clavien II (superficial wound infection) | 0 (Clavien III+) | Wound infection (n=1) | None | None | 1/39 (2.6%) |
| Dixon et al. (VOLTAIRE) | l-TAPP | 1/20 (5.0%) | 1 Clavien II (scrotal pain, treated conservatively) | 0 (Clavien III+) | Scrotal pain (n=1) | None | None | 0/20 (0%) |
| Valorenzos et al. (ROLAIS) | r-TAPP | 17/74 (23.0%) | Seroma, SSI, hematoma (numbers not stratified) | Bowel injury (n=1) | Seroma; SSI; hematoma; CPIP 5/74 | Bowel injury (1); Conversion (0) | None reported | 1/74 (1.4%) |
| Valorenzos et al. (ROLAIS) | l-TAPP | 27/65 (41.5%) | Seroma, SSI, hematoma (numbers not stratified) | Bowel injury (n=1); Conversion (n=1) | Seroma; SSI; hematoma; CPIP 8/65 | Bowel injury (1); Conversion (1) | None reported | 8/65 (12.3%) |

# **NR = not reported; SSI = surgical site infection; CPIP = chronic postoperative inguinal pain. Clavien–Dindo grading was only explicitly reported in Dixon et al. (VOLTAIRE), with no major (grade III+) events. For other trials, severity categories were not provided; bowel injury and conversion were considered proxies for major complications.**

# 
